# Silica encapsulation of ZnO nanoparticles reduces their toxicity for cumulus cell-oocyte-complex expansion

**DOI:** 10.1186/s12989-021-00424-z

**Published:** 2021-09-03

**Authors:** Antonella Camaioni, Micol Massimiani, Valentina Lacconi, Andrea Magrini, Antonietta Salustri, Georgios A. Sotiriou, Dilpreet Singh, Dimitrios Bitounis, Beatrice Bocca, Anna Pino, Flavia Barone, Valentina Prota, Ivo Iavicoli, Manuel Scimeca, Elena Bonanno, Flemming R. Cassee, Philip Demokritou, Antonio Pietroiusti, Luisa Campagnolo

**Affiliations:** 1 Department of Biomedicine and Prevention, University of Rome Tor Vergata, Via Montpellier 1, 00133 Rome, Italy; 2 Saint Camillus International University of Health Sciences, Via di Sant’Alessandro, 8, 00131 Rome, Italy; 3 Department of Microbiology, Tumor and Cell Biology, Karolinska Institute, SE-1, 71 77 Stockholm, Sweden; 4 Center for Nanotechnology and Nanotoxicology, Department of Environmental Health, T.H. Chan School of Public Health, Harvard University, 655 Huntington Ave, Boston, MA 02115 USA; 5 Department of Environment and Health, Istituto Superiore di Sanità, Viale Regina Elena 299, 00161 Rome, Italy; 6 Department of Public Health, Section of Occupational Medicine, University of Naples Federico II, Via S. Pansini 5, 80131 Naples, Italy; 7 Department of Experimental Medicine, University of Rome Tor Vergata, Via Montpellier 1, 00133 Rome, Italy; 8 Department of Inhalation Toxicology, National Institute for Public Health and Environment, 3721 MA Bilthoven, The Netherlands

**Keywords:** Zinc oxide nanoparticles, Titanium dioxide nanoparticles, Silica, Oocyte, Cumulus cells, Cumulus expansion, Extracellular matrix

## Abstract

**Background:**

Metal oxide nanoparticles (NPs) are increasingly used in many industrial and biomedical applications, hence their impact on occupational and public health has become a concern. In recent years, interest on the effect that exposure to NPs may exert on human reproduction has grown, however data are still scant. In the present work, we investigated whether different metal oxide NPs interfere with mouse cumulus cell-oocyte complex (COC) expansion.

**Methods:**

Mouse COCs from pre-ovulatory follicles were cultured in vitro in the presence of various concentrations of two types of TiO_2_ NPs (JRC NM-103 and NM-104) and four types of ZnO NPs (JRC NM-110, NM-111, and in-house prepared uncoated and SiO_2_-coated NPs) and the organization of a muco-elastic extracellular matrix by cumulus cells during the process named cumulus expansion was investigated.

**Results:**

We show that COC expansion was not affected by the presence of both types of TiO_2_ NPs at all tested doses, while ZnO NM-110 and NM-111 induced strong toxicity and inhibited COCs expansion at relatively low concentration. Medium conditioned by these NPs showed lower toxicity, suggesting that, beside ion release, inhibition of COC expansion also depends on NPs per se. To further elucidate this, we compared COC expansion in the presence of uncoated or SiO_2_-coated NPs. Differently from the uncoated NPs, SiO_2_-coated NPs underwent slower dissolution, were not internalized by the cells, and showed an overall lower toxicity. Gene expression analysis demonstrated that ZnO NPs, but not SiO_2_-coated ZnO NPs, affected the expression of genes fundamental for COC expansion. Dosimetry analysis revealed that the delivered-to-cell mass fractions for both NPs was very low.

**Conclusions:**

Altogether, these results suggest that chemical composition, dissolution, and cell internalization are all responsible for the adverse effects of the tested NPs and support the importance of a tailored, safer-by-design production of NPs to reduce toxicity.

**Supplementary Information:**

The online version contains supplementary material available at 10.1186/s12989-021-00424-z.

## Background

The great expansion of nanotechnology and increasing use of engineered nanoparticles (NPs) in many industrial and biomedical applications has raised concerns for human health and the environment. Over the last years, several studies have reported that most mammalian organs are targets for NPs independently of the route of exposure and, depending on specific NP physicochemical properties, NP-organ interaction may induce adverse effects [1, 2]. Indeed, pulmonary or oral administration of NPs results in accumulation of NP in most organs even far from the portal of entry, clearly indicating the ability of NPs to cross epithelial barriers and to reach the blood stream, through which they spread to peripheral sites [3–5]. This is further supported by the observation of NP deposition in almost all organs following intravenous injection [6]. It can then be speculated that high levels of NP deposition in highly vascularized organs, including the female reproductive apparatus, may pose concerns for potential consequent toxic effects [7].

Interestingly, over the last years, several studies have investigated the deposition and biological effect of a wide variety of nanoparticles on the male reproductive apparatus, while very few have studied the ability of a limited number of NP types to reach the female reproductive organs and eventually affect fertility [8, 9]. The female reproductive apparatus undergoes cyclic remodeling of its complex vascular system and represents one of the most sensitive systems in humans [10, 11]. The final number of female gametes, the oocytes, is definitively set around birth and fertility relies on it. Differently from the male gametes, the oocytes are not protected by anything similar to the blood-testis barrier, making them accessible to toxicants through the complex vascular bed of the organ, posing a serious concern for female fertility. Moreover, during the final stages of follicle growth preceding ovulation, vascular permeability increases, permitting the access of even large serum components [12] that, together with cumulus and oocyte products, allows the proper formation of follicular fluid that accumulates between cells and fills the antrum, the central cavity of Graafian preovulatory follicle. This physiological process may allow the arrival of potentially toxic substances present in blood close to the oocytes [13, 14]. Indeed, studies have reported that some NPs are able to access the female reproductive organs and possibly interfere with reproductive functions [15–17]; however, only a few reports have in depth proved the ability of NP to accumulate in the ovaries and affect follicular growth/oocyte maturation [17, 18]. Intravenous administration of silver NPs (AgNPs) resulted both in the reduced expression of genes critical for the development of primordial follicles (i.e. Foxo3A, Stella and Figla) and in the reduction of follicle number in the ovaries of treated mice [17]. Similarly, Gao et al. demonstrated that long-term oral exposure of female mice to titanium dioxide nanoparticles (TiO_2_NPs) leads to hormonal imbalance and disruption of ovarian gene expression, resulting in a reduction of pregnancy rate [15]. On the contrary, intra-peritoneal administration of gold nanoparticles (AuNPs) of different size (4.4, 22.5, 29.3, and 36.1 nm) did not induce any adverse effect in mouse ovaries [19]. Parallel to in vivo studies, in vitro models have been developed to evaluate the potential toxicity of NPs to female gametes [18, 20]. These models present the advantage to allow investigation of direct effect of toxicants on female fertility away from systemic influences and provide a sensitive tool to identify potential reproductive hazard. Cumulus-cell-oocyte complexes (COCs) can be isolated from pre-pubertal ovaries and cultured in vitro to study COC expansion and oocyte maturation, which are two processes propaedeutic to fertilization [21] and are considered sensitive toxicological parameters, since they can be impaired by toxicants at concentrations much lower than those affecting viability of mature oocytes [22]. Porcine COCs have been used to test toxicity of AgNPs and AuNPs. Interestingly, AgNPs demonstrated to accumulate mainly in the cumulus cells surrounding the oocyte and inhibit cumulus expansion and oocyte maturation, clearly indicating toxicity, while AuNPs were selectively taken up by the oocyte and did not show evident toxicity [18]. These data indicate the relevance of chemical composition of NPs in mediating their toxicity. Of note, the above mentioned in vitro models were designed to test either oocyte maturation or both COC expansion and oocyte maturation.

Despite the potential adverse effects of NPs on the female reproductive system, biomedical applications of specific NPs present several advantages for the identification and treatment of ovarian cancer [23], the most lethal gynecologic malignancy, so that design and production of NPs with reduced general toxicity in this context appear relevant. Recently, studies have focused at identifying the physicochemical properties potentially responsible for NP toxicity, leading to the synthesis of novel “safer-by-design” NPs, in which those properties are purposely modified [24, 25]. In this respect, over the last years, the role of dissolution of some metal oxide nanoparticles in mediating their toxicity has emerged [26] and efforts have been made to reduce it [25, 27–33]. In particular, NP dissolution has been demonstrated relevant for toxicity since the released ions appear responsible, at least in part, of the adverse effects.

**Fig. 1:**
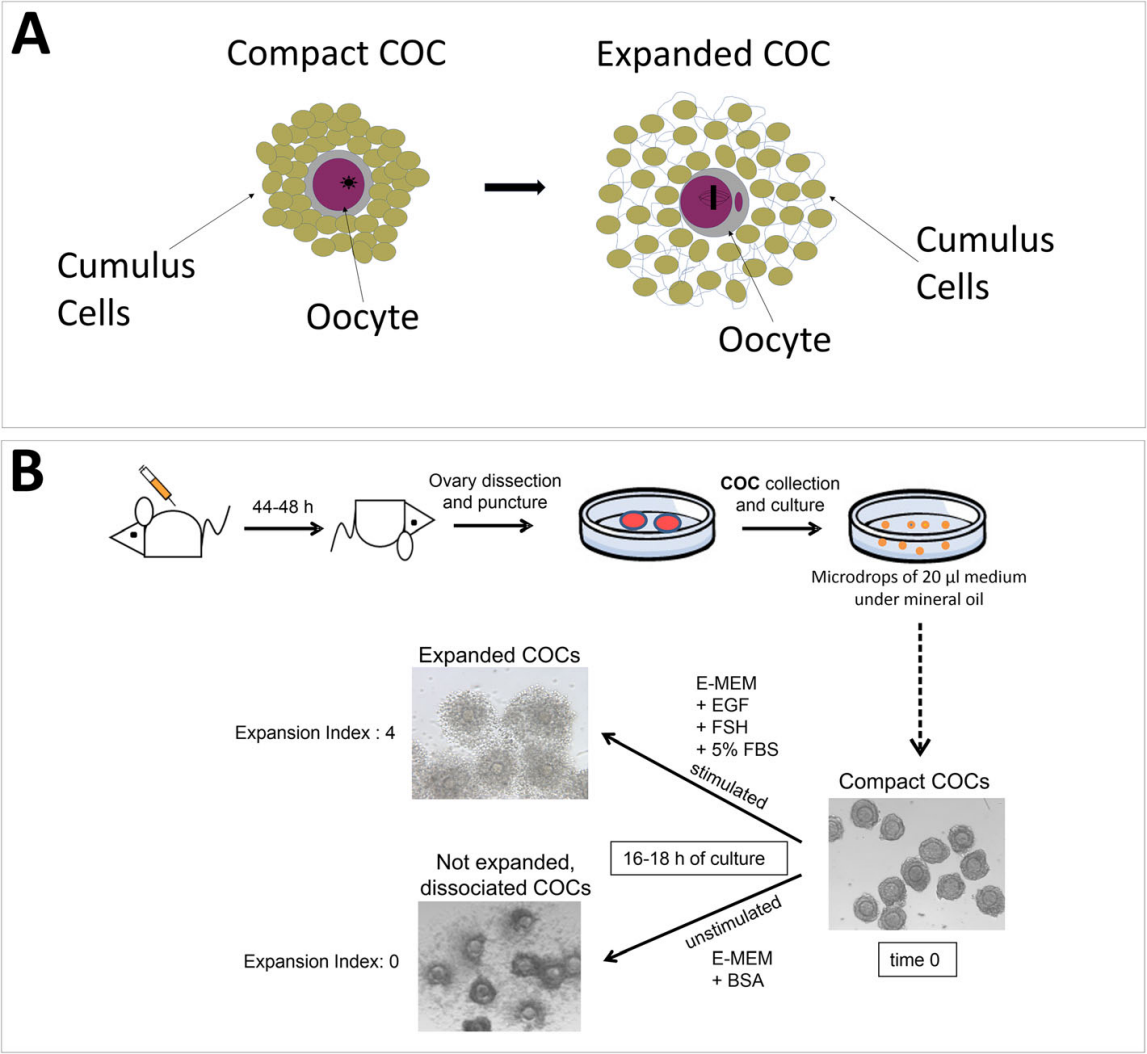
Schematic representation of the experimental settings. **a** Representation of the process of COC expansion upon meiosis resumption. **b** Schematic representation of the experimental setting used for the in vitro study

In the present study, we have used pre-ovulatory COCs isolated from pre-pubertal mouse ovaries (Fig. 1) and compared the ability of different types of metal oxide nanoparticles with tailored surface chemistry (namely TiO_2_, ZnO, and SiO_2_-coated ZnO nanoparticles) to interfere with the process of cumulus expansion in vitro, at both morphological and molecular level. Our results highlight the importance of different physicochemical characteristics in mediating the toxic effects, a relevant information for the safer-by-design production of NPs.

## Results

### NP synthesis and characterization

The TiO_2_ NPs used in this study were rutile TiO_2_ NM-103 and NM-104 obtained from the Repository for Representative Test Materials of the European Commission Joint Research Centre (JRC). A complete physicochemical characterization is available at the EU website [34]. Specifically, TEM analysis allowed defining the size of both types of particles, which was in the range of 22–26 ± 10 nm, and also identified that both NP powders were highly aggregated, with aggregates in the size range of 20–500 nm. Energy Dispersive X-ray Spectroscopy (EDX) analysis allowed to identify the presence of contaminants, including Al, Si, Fe and S. Suspension of NPs in culture medium was stable, and no dissolution was observed in three different media. Two of the tested ZnO NPs (NM-110 and NM-111) were also obtained from the JRC and their physicochemical characterization is available at the EU website [35]. X-ray Diffraction (XRD) analysis identified a crystal size of 41.5 and 33.8 nm for NM-110 and NM-111, respectively. Dissolution was demonstrated for NM-110 in DI water. NM-111 was characterized by the presence of a triethoxycaprylsilane coating, which made the particles hydrophobic and difficult to disperse for dissolution studies. The other two ZnO NPs used in this study were prepared in-house using flame spray pyrolysis in the Harvard Versatile Engineered Nanomaterial Generation System (VENGES) as described previously and characterized in detail for their physicochemical and morphological properties [25, 36–38]. These NPs consisted of pure uncoated ZnO nanorods (uZnO NPs) with an average crystal size of 29 nm and specific surface area (SSA) of 41 m^2^/g, and SiO_2_-coated ZnO nanorods (SiO_2_ZnO NPs) with an average crystal size of 28 nm and SSA of 55m^2^/g, which were hermetically and homogenously encapsulated in a ~ 5 nm amorphous silica shell (Supplemental Fig. 1). Both uZnO and SiO_2_ZnO nanorods had an average aspect ratio of 3:1 (ranging from 2:1 to 8:1). The silica coating efficiency on the ZnO nanorods was 95%.

### DLS characterization of uZnO and SiO_2_ZnO NPs

Dynamic light scattering (DLS) analysis of the dispersions in ddH_2_O highlighted a lower hydrodynamic diameter size of the SiO_2_ZnO NPs (Z-ave = 162.1 ± 7.35 nm) compared to the uncoated uZnO NPs (Z-ave = 235.5 ± 16.48 nm), as reported in Table 1. The polydispersity index (PDI) values of 0.123 ± 0.03 and 0.161 ± 0.02, respectively, reflected that both samples were monodispersed in water. After dilution in complete culture medium, a marked change in Z-ave and PDI values was observed for both NPs concentrations (5 and 10 μg/mL) and for the different time of exposure. In particular, the decrease of Z-ave was dependent on the time of exposure only for the SiO_2_ZnO NPs, while the strong increase of PDI was not correlated to experimental conditions and was displayed by both uncoated and coated NPs.

**Table 1:** Z-average and PDI values of SiO_2_ ZnO NPs and uZnO NPs in H_2_O and in culture medium (EMEM)

			SiO_2_ ZnO NPs		uZnO NPs	
Medium	Time (h)	Conc. (μg/ml)	Z - ave ± sd^a^ (nm)	PDI ± sd	Z ave ± sd^b^ (nm)	PDI ± sd
ddH2O	0	1000	162.10 ± 7.35	0.123 ± 0.03	235.53 ± 16.48	0.161 ± 0.02
EMEM	0	5	65.12 ± 1.39	0.695 ± 0.01	15.58 ± 0.66	0.413 ± 0.01
EMEM	0	10	91.21 ± 8.18	0.567 ± 0.09	27.51 ± 7.17	0.738 ± 0.14
EMEM	3.5	5	25.97 ± 2.29	0.815 ± 0.07	21.03 ± 5.70	0.602 ± 0.18
EMEM	3.5	10	52.19 ± 8.81	0.761 ± 0.11	23.20 ± 8.86	0.675 ± 0.28
EMEM	16	5	19.27 ± 0.33	0.470 ± 0.004	19.80 ± 4.59	0.580 ± 0.16
EMEM	16	10	23.70 ± 2.79	0.747 ± 0.09	20.67 ± 0.68	0.574 ± 0.06

^a^The reported values are the mean of two independent experiments

^b^The reported values are the mean of three independent experiments

### TiO_2_ NPs do not affect COC expansion

**Fig. 2:**
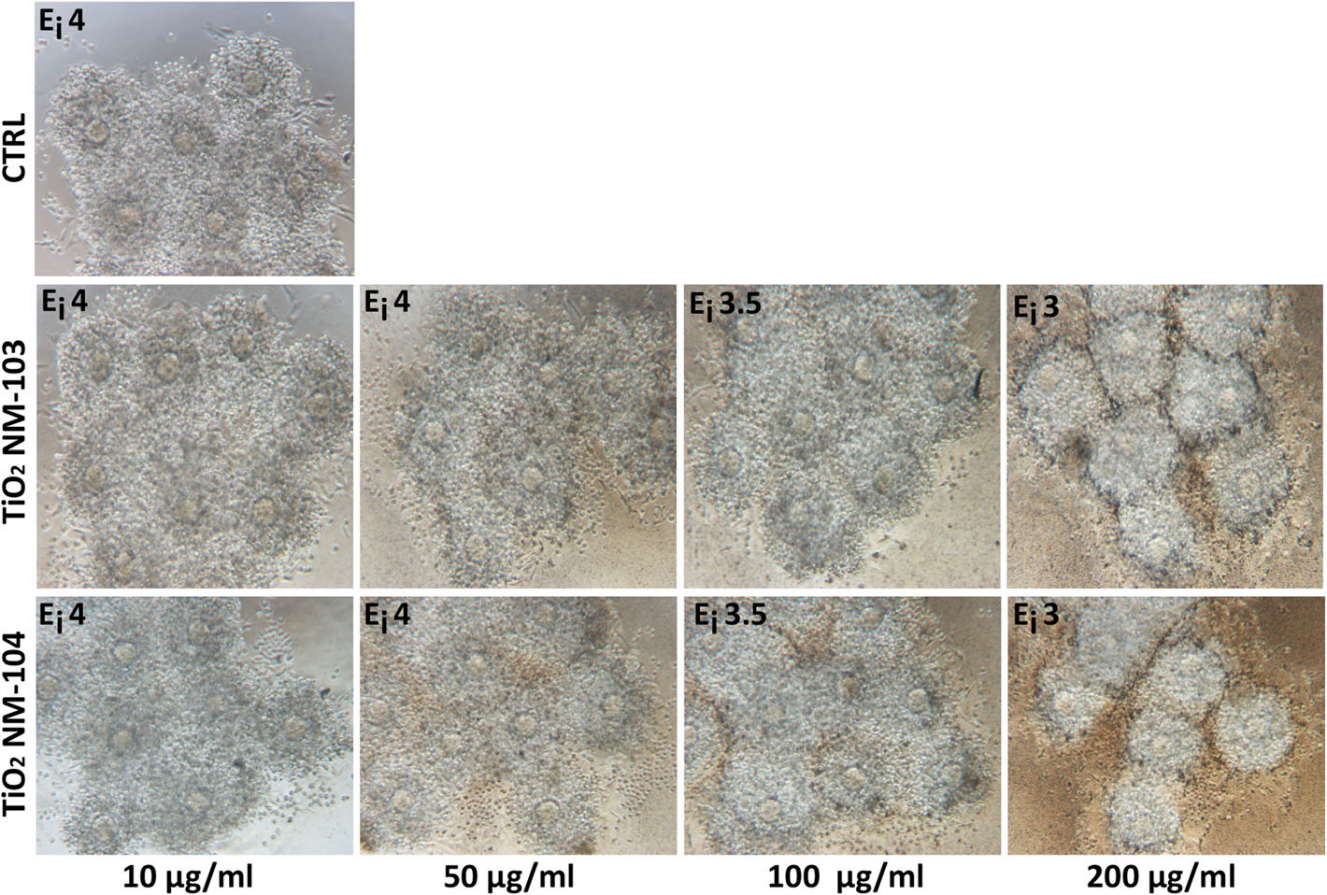
COC expansion in the presence of increasing concentrations of TiO_2_ NPs. COCs cultured in increasing concentrations of TiO_2_ NPs were evaluated in comparison to the control culture (CTRL), in which COCs were not exposed to NPs. In all samples, the extent of COC expansion was assessed at the end of the culture time (16 h) and expressed as expansion index (E_i_)

To investigate if NPs may demonstrate adverse effects on the organization of the cumulus extracellular matrix in vitro, interfering with cumulus cell-oocyte complex (COC) expansion, isolated COCs were cultured in groups of 10 into microdrops of medium containing increasing concentrations of TiO_2_ NPs (NM-103 and NM-104), and after 16 h cumulus expansion was evaluated. As shown in Fig. 2, the typical expanded morphology of COCs was observed in the control cultures and the expansion index was evaluated as 4 (E_i_ 4). Similar indexes (E_i_ 4 and E_i_ 3.5) were attributed to COCs cultured in the presence of both NM-103 and NM-104 at concentrations of 10, 50 and 100 μg/ml. Interestingly, at the very high dose of 200 μg/ml expansion was still appreciated, although the expansion index attributed to this COCs was slightly decreased and evaluated as E_i_ 3 (Fig. 2). These results suggest that TiO_2_ NPs are very little toxic to COCs and do not impair the process of extracellular matrix deposition and expansion.

### ZnO NM-110 and NM-111 strongly affect COC expansion

**Fig. 3:**
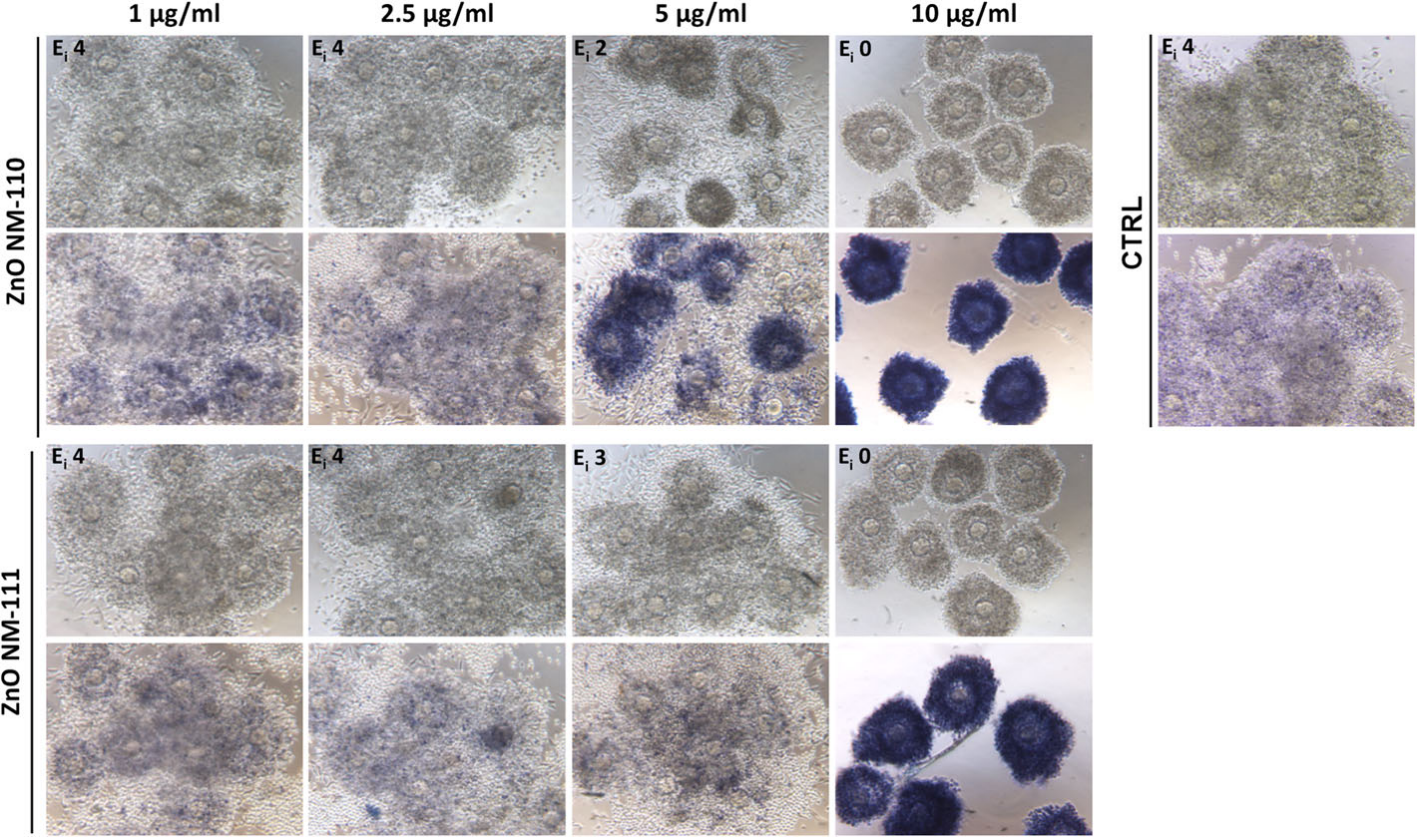
COC expansion in the presence of increasing concentrations of ZnO NPs NM-110 and NM-11. For each type of NPs, the first lane of micrographs shows COC morphology after 16 h of culture; at that time, the trypan blue staining was performed to identify dead cells, as shown in the second lane. Control culture (CTRL) without the addition of NPs was carried out in parallel. In all samples, the extent of COC expansion was assessed at the end of the culture time (16 h) and expressed as expansion index (E_i_)

To analyze whether NPs with different physicochemical properties may have different effects on COC expansion, COCs were cultured for 16 h in the presence of increasing concentrations of ZnO NM-110 and NM-111. As shown in Fig. 3, COCs incubated with 5 μg/ml of NM-110 demonstrated reduced expansion, with an assigned expansion index of 2. In this condition, trypan blue staining highlighted the presence of dead cumulus cells surrounding the oocyte. This effect was exacerbated when COCs were cultured in the presence of 10 μg/ml, a concentration at which expansion was completely impaired (E_i_ 0) and all cumulus cells appeared heavily stained by trypan blue. Interestingly, NM-111 seemed to be less toxic than NM-110, since a level of expansion, rated as 3, was still present in the microdrops containing 5 μg/ml of the NPs. However, expansion was completely inhibited at 10 μg/ml and trypan blue stained the whole COCs, similarly to what observed for NM-110 at this same concentration (Fig. 3).

**Fig. 4:**
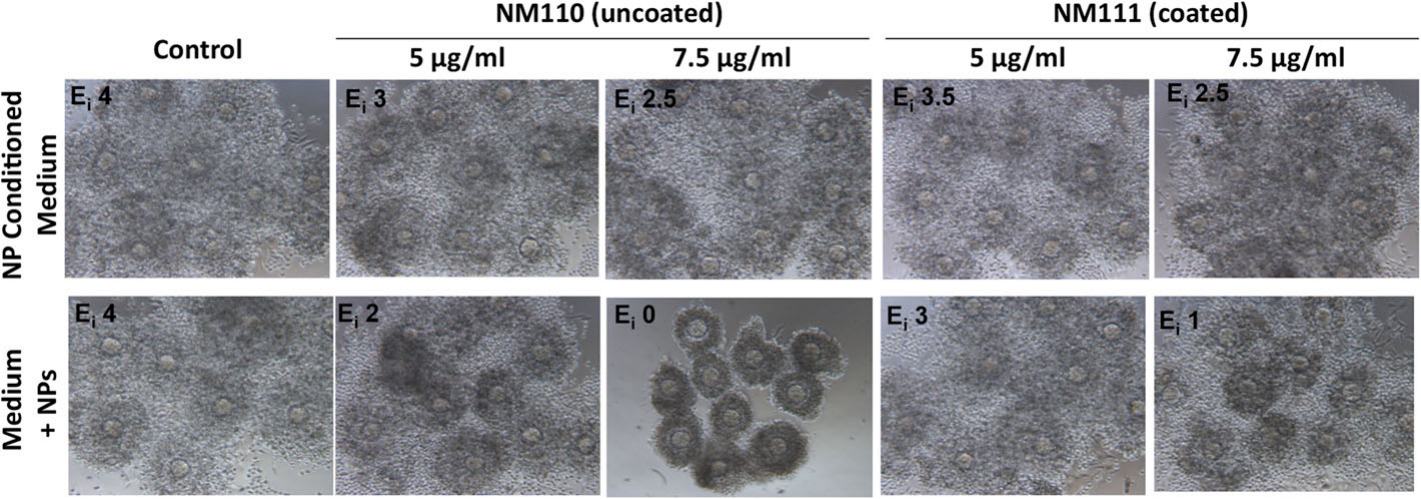
Effect of NPs and NP-conditioned media on COC expansion. After incubation of NPs for 16 h in culture medium, the resulting conditioned medium, deprived of NPs through centrifugation, was used to culture COCs (upper panel). In parallel, COCs cultures were performed in the presence of the indicated NPs (lower panel). In all samples, the extent of COC expansion was assessed after 16 h incubation, and expressed as expansion index (E_i_)

The influence of the potential release of Zn^2+^ ions in mediating toxicity was investigated by culturing the COCs in medium conditioned for 16 h by the two types of ZnO NPs at two different concentrations. As reported in Fig. 4, COCs cultured in the presence of NP conditioned medium showed expansion indexes lower than those of control cultures, but higher than those recorded for COCs cultured with NPs at the same corresponding concentrations, suggesting that inhibition of COC expansion is only in part caused by Zn^2+^ ions released into the medium.

### Silica encapsulation of ZnO NPs reduces adverse effects on COC expansion

**Fig. 5:**
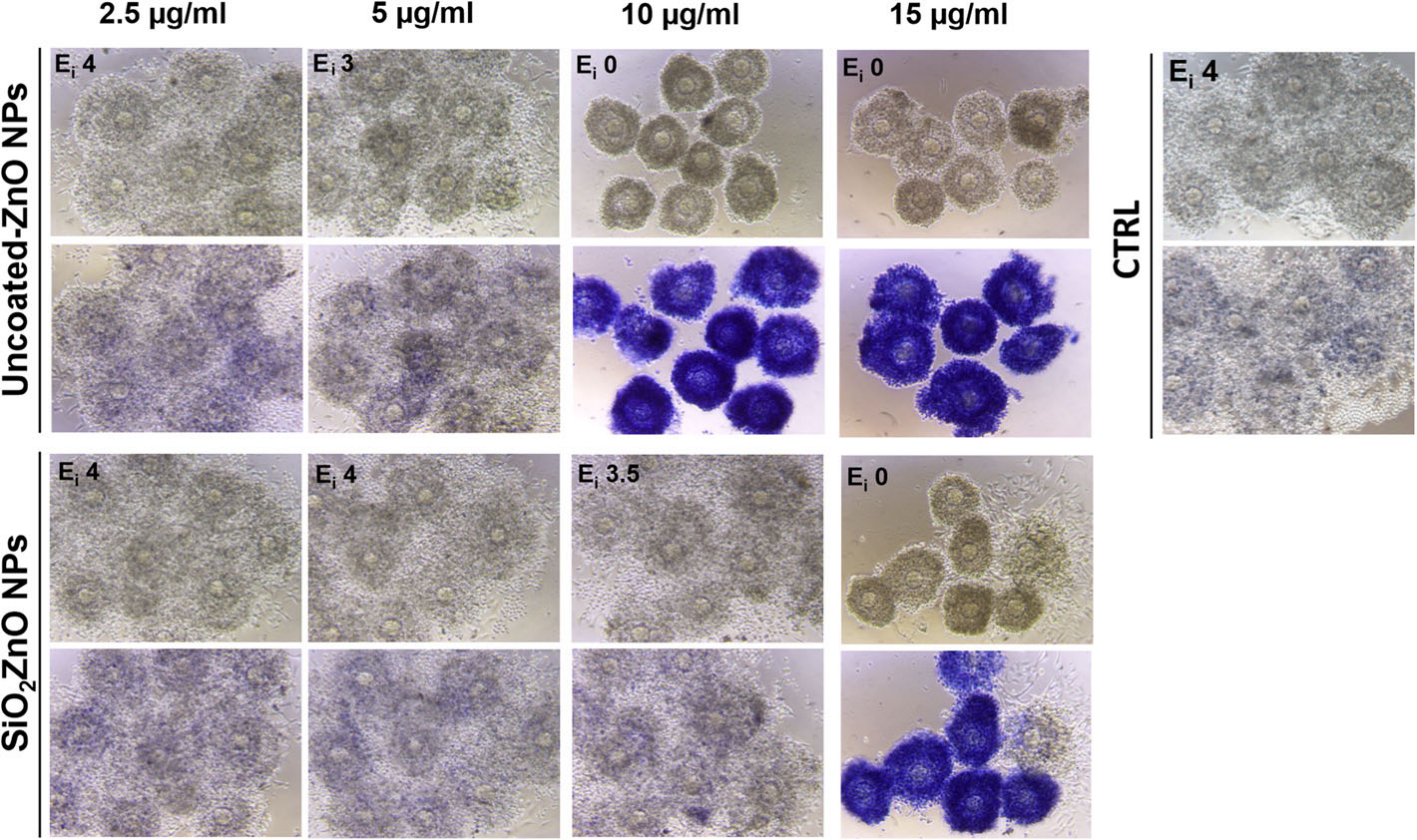
COC expansion in the presence of increasing concentrations of the in-house prepared uZnO and SiO_2_ZnO NPs. For each type of NP, the first lane of micrographs shows the COC morphology after 16 h of culture; at that time, trypan blue staining was performed to identify dead cells, as shown in the second lane. Control culture (CTRL) without NPs was carried out in parallel. In all samples, the extent of COC expansion was assessed at the end of the culture (16 h) and expressed as expansion index (E_i_)

To further investigate the role of released Zn^2+^ ions following NP dissolution in mediating toxicity, we compared the ability of in-house prepared uZnO NPs and SiO_2_ZnO NPs to interfere with COC expansion. At the concentration of 5 μg/ml both NPs showed very little or no toxicity. However, at the concentration of 10 μg/ml the uncoated NPs completely inhibited expansion and induced massive cell death as demonstrated by the trypan blue staining, while the SiO_2_ZnO NPs did not affect COC expansion nor induced any cell death (Fig. 5), clearly indicating that the presence of the silica shell was limiting toxicity. Nevertheless, by increasing the concentration to 15 μg/ml, COC expansion was also inhibited by the SiO_2_ZnO NPs. We then compared toxicity of the two NPs on the basis of administered mass fraction (f_D_) delivered on the surface of COC as a function of time, following a method we previously described [39]. The effective densities of uZnO and SiO_2_ZnO NPs had been formerly found to be 1.485 and 1.655 g/mL, respectively [40]. As shown in Table 1, the hydrodynamic size (expressed as the Z-average) of both types of NPs depended on their starting concentration in the growth medium, ranging from 16 to 91 nm. Given an approximate medium viscosity and density of 0.00074 Pa s and 1.00 g/ml, respectively, and in combination with the dissolution rate of the uZnO and SiO_2_ZnO NPs, it was possible to calculate their respective f_D_, as presented in Supplemental Fig. 2. Due to small hydrodynamic sizes and light densities, once added in growth medium, both uZnO and SiO_2_ZnO NPs presented with very low f_D_. In brief, for both types of particles, less than 1% of the administered mass is expected to deposit on COCs during the 16 h of exposure.

**Fig. 6:**
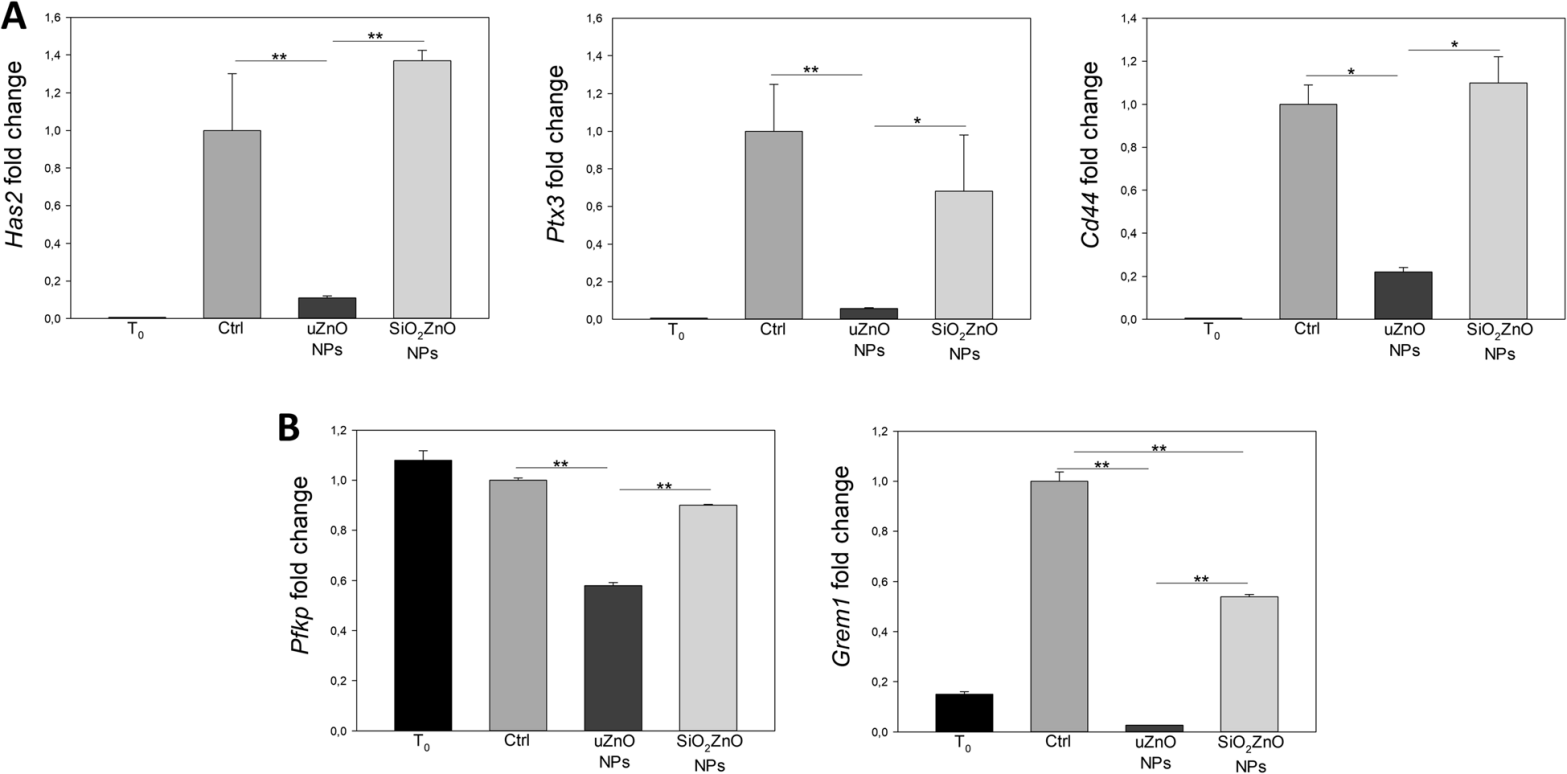
Quantitative analysis for the expression of genes involved in cumulus expansion, metabolism and cumulus response to oocyte signals. **A** Quantitative RT-PCR data for *Has2*, *Ptx3* and *Cd44* in COCs at the beginning (T_0_) and after 3.5 h of culture without nanoparticles (Ctrl) and in presence of 10 μg/ml of uZnO and SiO_2_ZnO NPs, respectively. **B** Quantitative RT-PCR data for *Pfkp* and *Grem1* in COCs at the beginning (T_0_) and after 3.5 h of culture without nanoparticles (Ctrl) and in presence of 10 μg/ml of uZnO and SiO_2_ZnO NPs, respectively. *P* ≤ 0.05 is indicated by *, *P* ≤ 0.001 is indicated by **. With the exception of *Pfkp*, T_0_ is always significantly different from Ctrl with *P* ≤ 0.001, with the exception of T_0_ vs Ctrl for *Has2*, where *P* ≤ 0.05

To assess the molecular mechanisms regulating the different response to the two types of nanoparticles, we performed gene expression analysis for genes involved in the formation and organization of the muco-elastic extracellular matrix by cumulus cells, in oocyte metabolism and cumulus response to oocyte signals. Since it is known from previous data [41] that the in vitro stimulus, follicle stimulating hormone (FSH) and epidermal growth factor (EGF), that promotes activation of gene expression by cumulus cells acts very early, during the first 1–2 h, we performed gene expression analysis by real-time RT-PCR on COCs cultured for 3.5 h (Fig. 6). The results clearly show that exposure of the cells to 10 μg/ml of the uncoated NPs strongly inhibited the expression of hyaluronan synthase 2 (*Has2)*, Pentraxin 3 (*Ptx3)* and hyaluronan receptor *Cd44*, implicated in hyaluronan synthesis, stabilization of the expanded matrix and cell adhesion to hyaluronan, respectively. On the contrary, COCs treated with the silica coated NPs did not show significant differences compared to control, indicating that the presence of the silica shell prevents the adverse effects of the ZnONPs (Fig. 6A). We also investigated the expression of Phosphofructokinase, platelet (*Pfkp*), one of the key enzymes in glycolysis, and of Gremlin 1 (*Grem1*) a downstream target of GDF9. uZnO significantly reduced the expression of *Pfkp* and almost completely abrogated the expression of *Grem1* (Fig. 6B). On the contrary, SiO_2_ZnO NPs had no effect on the expression of *Pfkp*, while significantly reduced the expression of *Grem1* compared to control, although the effect was much less dramatic and significantly different from that of uZnO NPs (Fig. 6 B).

Indeed, morphological analysis of COCs prior to RNA extraction confirmed the molecular data, as control cultures and COCs exposed to SiO_2_ZnO NPs showed a comparable morphology, which was different from that of COCs cultured in the presence of uZnO NPs (Supplemental Fig. 3).

**Fig. 7:**
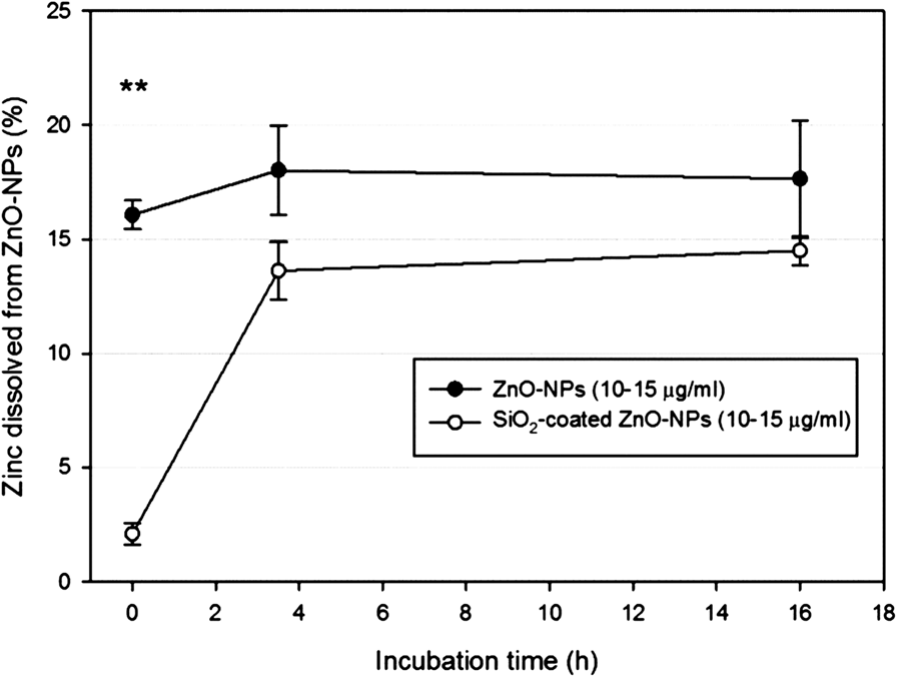
Analysis of Zn^2+^ ions released during the culture. ICP-MS analysis of Zn^2+^ ions present in culture medium after 0, 3.5 and 16 h of incubation of uZnO and SiO_2_ZnO NPs, both at concentration of 10 μg/ml. The results are presented as percentage of the starting concentration (mean ± sd)

To evaluate if the release of Zn^2+^ ions was responsible for the toxicity of the uZnO NPs and hence the reduced toxicity of the SiO_2_ZnO NPs, the amount of released ions was quantitated by inductively coupled plasma mass spectrometry (ICP-MS) in the culture medium at the beginning and after 3.5 and 16 h of incubation, following removal of undissolved nanoparticles. Results indicate that the uZnO NPs rapidly dissolved Zn^2+^ ions in the culture medium (Fig. 7), while the SiO_2_ZnO NPs underwent slower dissolution and reached levels similar to those of the uZnO NPs only after 3.5 h of incubation.

### SiO_2_ZnO and uZnO NPs show different uptake in COCs

**Fig. 8:**
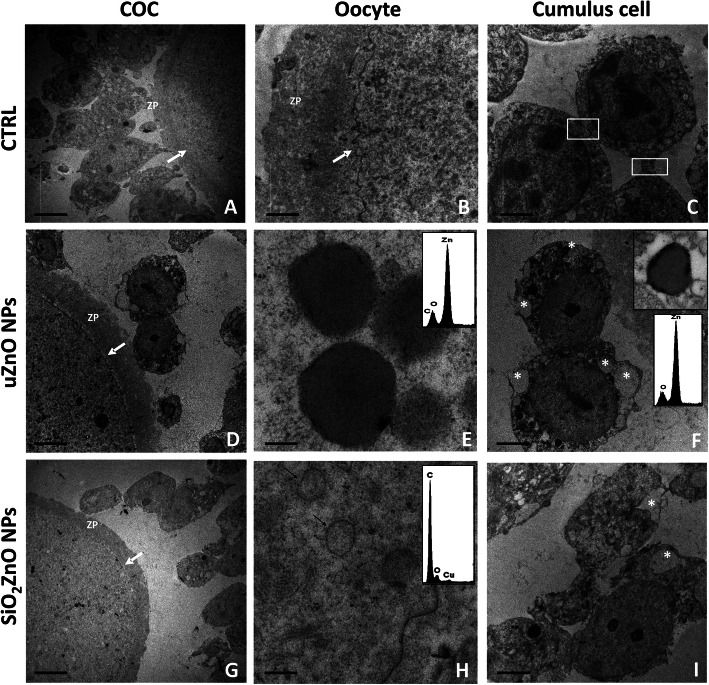
TEM analysis of COCs cultured for 3.5 h in the absence and in the presence of either uZnO or SiO_2_ZnO. **A** COC in control cultures. Scale bar 20 μm. **B** Detail of oocyte from a control culture. Scale bar 2 μm. **C** Cumulus cells from control culture, which appear well preserved and connected by desmosomal structures (white boxes). Scale bar 5 μm. **D** COC cultured with uZnO NPs. Scale bar 20 μm. **E** Detail of an oocyte treated with uZnO NPs, which shows lysosomes containing electrondense particles with peripheral sublocalization. EDX analysis confirmed the content of Zn and O (EDX spectrum). Scale bar 0.2 μm. **F** Cumulus cells treated with uZnO NPs display cytoplasmic vacuolation (asterisks) and several Zn and O containing lysosomes (inset) (EDX spectrum). Scale bar 5 μm. **G** COC cultured with SiO_2_ZnO NPs. Scale bar 20 μm. **H** Detail of an oocyte cultured with SiO_2_ZnO NPs; no Zn containing lysosomes are identified. Scale bar 0.5 μm. **I** Cumulus cells treated with SiO_2_ZnO NPs display moderate signs of degeneration such as cytoplasm vacuolation (asterisks). No lysosomes containing electrondense particles are identified. Scale bar 5 μm. ZP: zona pellucida; white arrows: oocyte plasma membrane; black arrows: lysosomes

To verify the possible intracellular localization of ZnO NPs in cumulus cells and the oocyte, we performed TEM analysis of COCs cultured for 3.5 h without (control) or with uZnO and SiO_2_ZnO NPs, both present at 10 μg/ml (Fig. 8). The analysis demonstrated that both cumulus cells and oocytes in the control samples showed no evidence of cell degeneration (Fig. 8 a-c). Cumulus cells were connected to each other by desmosomal structures and showed, in addition, interactions with the oocyte (Fig. 8c). On the contrary, after 3.5 h of culture in the presence of uZnO NPs, cumulus cells showed sporadic contacts with the oocyte and evident signs of cell degeneration, such as cytoplasmic vacuolization, alteration of plasma membrane and mitochondria swelling (Fig. 8D-F). Moreover, the presence of cell debris suggestive of apoptotic bodies were observed in different areas of the sample (Fig. 8). Approximately 30% of the observed cumulus cells presented lysosomal structures containing electron-dense particles, and similar structures were observed in the oocyte (Fig. 8E,F). The chemical composition of these particles was assessed by Energy Dispersive X-ray Spectroscopy (EDX) analysis (Fig. 8E, F), which confirmed the content of Zn and O in these areas, suggesting that the observed particles could be the ZnO NPs, intact/partly dissolved and/or the released zinc ions. Interestingly, when COCs were cultured in the presence of SiO_2_ZnO NPs, no electron-dense zinc containing particles were observed inside both the cumulus cells and the oocytes, as confirmed by EDX analysis, and the oocyte did not show any sign of degeneration (Fig. 8G-I).

## Discussion

Over the last decades, an increasing number of studies has focused on the impact of newly developed materials on germ cells and the reproductive apparatus. This great interest relies on two main reasons. On one side, fertility problems are becoming a serious concern and more couples turn to assisted reproductive technologies, with environmental and occupational factors being recognized to often have a causal role. On the other side, germ cells are particularly sensitive to xenobiotics, thus allowing to identify potential subtle toxicity of compounds. Here we show that COC expansion is a valuable system to identify physicochemical characteristics mediating toxicity of specific engineered NPs. In particular, we show the influence of chemical composition and dissolution of NPs on the organization of the muco-elastic extracellular matrix of the COCs, namely cumulus expansion, and on the expression of metabolic enzymes and of oocyte-induced cumulus factors, all fundamental to allow fertilization of the oocyte [21, 42–47].

Our results demonstrate that TiO_2_ NPs show no overt effects on cumulus expansion. We have used two types of TiO2 NPs, both rutile and with similar size, however NM-103 was hydrophobic and NM-104 was hydrophilic. Nevertheless, we did not observe any difference with respect to inhibition of COC expansion for both formulations, even at doses as high as 200 μg/ml. Our results suggest that TiO_2_ may have very little impact on COC expansion and consequently on oocyte fecundability. The observed low toxicity of TiO2 NP may depend on several factors, including chemical composition, levels of internalization and reduced dissolution. Our results are in agreement with what has been previously demonstrated in vivo after oral short-term and acute administration [16, 48], while appear in contrast with one study reporting adverse effects of TiO2 NPs on ovarian cells after long term intragastric treatment [15]. However, in this study, it was not clear which type of ovarian cells the authors refer to and direct interaction between TiO_2_ NPs and ovarian follicles was not clearly demonstrated. Since the authors reported significantly different levels of gonadotropins, the observed effects on fertility might be due, at least in part, to NP-induced hormonal imbalance [15]. Our proposed in vitro model allows discriminating between the adverse effects arising from direct interaction between NPs and pre-ovulatory COCs and potential systemic effects of NPs.

Interestingly, when COCs were cultured in the presence of metallic NPs of a different chemical composition, i.e. ZnO NPs, we observed a strong reduction of COC expansion, even at doses as low as 5 μg/ml. This different behavior in comparison to the TiO_2_ is probably related to the different chemical composition, although it cannot be excluded that NP shape may also play a role. Since ZnO NPs are known to undergo rapid dissolution, we compared the effect of fast dissolving uncoated ZnO NPs (i.e. ZnO NM-110 and in-house prepared uZnO NPs) to that of coated ZnO NPs (i.e. triethoxycarpryl silane-coated NM-111 and SiO_2_ZnO NPs). As expected, the coated NPs displayed lower level of toxicity, demonstrating that dissolution plays an important role in mediating toxicity. Indeed, the SiO_2_ZnO NPs showed the lowest toxicity among all the ZnO NPs, demonstrating the advantage of stable encapsulation of ZnO NPs into a nano-silica shell for increasing biocompatibility, while not altering the optoelectronic properties of ZnO NPs [25]. Nevertheless, the observed toxicity of ZnO NPs could not be solely ascribed to dissolution, at least for the NM-110 and NM-111NPs, since the use of medium conditioned by these NPs did not reproduce the observed effect on cumulus expansion after culture with the NPs themselves at the same concentrations, indicating that other factors are at play to induce toxicity. Nanoparticle-cell interaction and nanoparticle internalization are possibly other parameters to control. Our ultrastructural analysis associated to EDX, identified the presence of Zn-containing electron-dense particles in both cumulus cells and the oocyte of COCs cultured for 3.5 h with uZnO NPs. These particles may have crossed the zona pellucida due to their small size (28 nm) and their rapid dissolution that further reduces the size, and after internalized by the oocyte through endocytosis. Indeed, virus in the range of 20–30 nm, as well as gold nanoparticles have been reported to cross the zona pellucida [18, 49]. The observed particles were evident in lysosomes of both cumulus cells and oocytes, with an unusual peripheral sublocalization, close to the lysosomal membrane, which might suggest rapid uptake in this organelle and/or organelle damage, with loss of membrane integrity. Although Zn-containing particles were localized in both cumulus cells and the oocyte, only the cumulus cells presented evident signs of cell degeneration, while the oocytes did not show apparent ultrastructural alterations. Encapsulation of ZnO NPs into a silica shell possibly prevented or reduced cellular interaction and uptake, as no particles containing Zn or Si could be identified in either cumulus cells or the oocytes following the TEM-EDX analysis, suggesting that after 3.5 h of culture NPs are not internalized or their cellular levels are too low to be detected. Interestingly, the dose of uZnO and SiO_2_ZnO NPs delivered to the cells appeared to be very low (less than 1% as calculated by the dosimetric analysis), suggesting that toxicity is primarily driven by the release of Zn^2+^ ions, which would further support the relevance of NP encapsulation into silica to increase biocompatibility. Indeed, many studies have reported biocompatibility of silica in several organs [7, 50]. A different dissolution rate during the first 3.5 h for uZnO and SiO_2_ZnO NPs was confirmed by ICP-MS analysis, with the uncoated particles reaching the maximal dissolution immediately after suspension and the coated particles slowly releasing low levels of Zn^2+^ ions during the first 1–2 h. This temporal window is of pivotal importance for the expansion of cumulus cells, as the stimulus exerted by FSH is only needed for the first 2 h in order to stimulate maximal synthesis of HA [41]. Indeed, zinc is an essential trace element, and several data demonstrate that regulation of free zinc levels is essential for oocyte maturation and oocyte arrest in metaphase II [51–53]. Lisle et al. demonstrated that cumulus cells timely regulate the levels of free zinc in the oocyte during maturation, and that these cells maintain low the levels of free zinc in the oocyte before ovulation. Indeed, in vitro studies in farming animals demonstrated that low concentrations of Zn (0.7, 1.1 and 1.5 μg/ml) reduce apoptosis, promote DNA integrity and SOD activity in cumulus cells, and improve developmental capacity of the oocyte [54]. Stephenson and Brackett [55] demonstrated that zinc at 10 μg/ml inhibits in vitro maturation of bovine oocytes. Our results support the relevance of zinc concentration. In our experimental settings, the concentrations of uZnO NPs and SiO_2_ZnO NPs ranged between 2.5 and 10 μg/ml. ICP-MS results show a rapid increase of released zinc ions which is likely responsible, at least in part, for the toxicity observed. To further support the relevance of particle dissolution rate within this initial temporal window, we performed gene expression analysis for genes directly involved in the organization of the cumulus extracellular matrix. Our results clearly show that uZnO NPs significantly inhibited the up regulation of *Has2*, *Ptx3* and *Cd44* observed during the first 3.5 h of culture in the absence of NPs. Interestingly, gene expression differences correlated with morphological differences, since COCs cultured with uncoated NPs showed no sign of cumulus cell spreading, differently from what observed in control and coated NPs cultures, and were greatly fragile with the oocyte easily detaching from the cumulus cell layers. These results suggest that, although massive cell death and inhibition of cumulus expansion become evident after 16 h of culture, cell alterations and disorganization of cumulus matrix appear already at earlier stages. In addition, our data clearly show that specific NPs tested in vitro on the process of cumulus expansion showed a toxicity associated with an altered expression of genes involved in the production of components of the cumulus extracellular matrix and their assembly. To further elucidate the potential impact of the tested NP on oocyte health, we investigated whether exposure to uZnO NPs and SiO_2_ZnO NPs may affect cumulus cell glycolysis, on which the oocyte relies for its metabolic demand [56]. We observed that uZnO NPs significantly reduced the expression of phosphofructokinase (Pfkp), one of the key enzymes in the glycolytic chain. Interestingly, association between Pfkp levels and pregnancy outcome has been proposed, suggesting that Pfkp expression in cumulus cells may allow to identify oocytes with higher developmental capacity [57]. Indeed, increased glucose consumption was demonstrated in in vitro matured COCs that could then be fertilized compared to those that could not [58]. We also investigated the expression of Grem1, a downstream target of oocyte-derived GDF9; it has been proposed that the expression levels of GDF9 downstream genes in cumulus cells may predict oocyte health [59]. Interestingly, after 3.5 h of culture, when cumulus cell death was not evident yet, uZnO NPs dramatically reduced the expression of Grem1, while the SiO_2_ZnO NPs demonstrated a much lower effect, although they also significantly reduced the expression of Grem1 compared to the control. Our study presents some limitations. First, our in vitro model relies on pre-ovulatory COCs, in which oocyte maturation can be hardly assessed, while most of the published literature has evaluated the influence of cumulus cells on oocyte using post-ovulatory COCs. Therefore, our results do not allow to directly correlate COC expansion with oocyte developmental competence, although the evaluation of metabolic markers in cumulus cells gives an indirect assessment of oocyte quality. Moreover, it should be considered that in vitro studies may not be directly translated to in vivo conditions. The concentrations used in this study were chosen in order to identify the highest non-effective and the lowest effective doses and compare them among the different nanoparticles used. These doses hardly represent a potential real world exposure in vivo, since several factors (e.g., corona formation in blood, extent of organ vascularization, acute versus chronic exposure) may largely affect the amounts of NPs reaching the organ and cannot be adequately reproduced in any in vitro study.

It would be of interest to investigate further this behavior, by setting in vivo experiments in order to evaluate the impact of the different NPs on reproductive performances, since it is known that an adequate cumulus expansion is the prerequisite for a successful fertilization.

## Conclusions

In conclusion, our results demonstrate that metal oxide nanoparticles with different physicochemical properties differently affect the expansion of COCs and consequently the ability of the oocyte to be fertilized. Many factors appear to mediate toxicity. Nanoparticle dissolution and consequent ion release, cellular internalization and chemical composition may all contribute to toxicity. Moreover, COC cultures may represent a valuable in vitro tool to screen for toxicity of NPs, and to discriminate among the physicochemical properties driving the adverse effect; further confirmatory studies using post-ovulatory COCs may strengthen the predictivity of the model. These results further support the importance of the safer-by-design approach for the production of NPs in which the physicochemical characteristics mediating toxicity are controlled, while retaining the advantageous properties [24, 25, 30, 32, 33, 60]. In addition, our studies suggest that specific metallic NPs might represent a threat for female fertility; however in vivo studies should be designed following a relevant exposure scenario, with a particular attention to exposure concentrations, route and chronicity.

## Methods

### Nanoparticle synthesis and characterization

Rutile TiO_2_ nanoparticles NM-103 and NM-104, and ZnO nanoparticles NM-110 and NM-111 were obtained from the JRC repository. Detailed physicochemical characterization of the particles used in this study is provided in the JRC reports [61, 62]. uZnO and SiO_2_ZnO NPs were synthesized using flame spray pyrolysis in the Harvard-based Versatile Engineered Nanomaterial Generation System (VENGES) as previously described by Sotiriou et al. [25]. Detailed physicochemical and morphological characterization of the uZnO and SiO_2_ZnO NPs was performed using state-of-the art analytical methods such as transmission electron microscope (TEM, for size and morphology), x-ray diffraction (XRD, to assess crystal structure and size), x-ray photoelectron spectroscopy (XPS, to assess silica coating efficiency) and Brunauer–Emmett–Teller (BET, to measure specific surface area), also previously described in detail [25].

### Cell culture system

Immature 18–21 day-old female Swiss CD-1 mice were injected with 5 IU of pregnant mares’ serum gonadotropin in 0.1 ml of physiological saline and sacrificed 44–48 h later by cervical dislocation. COCs from pre-ovulatory (Graafian) follicles were collected by puncturing the dissected ovaries. COCs consist of several layers of compacted follicular cells (cumulus cells) surrounding the oocyte. Cumuli were cultured for either 3.5 or 16–18 h in microdrops (20 μl) of medium overlaid with mineral oil (Sigma Aldrich) to avoid evaporation, at 37 °C in a humidified atmosphere of 5% CO_2_. The culture medium consisted of Minimum Essential Medium with Earle’s salts (EMEM) supplemented with 3 mM glutamine, 0.3 mM pyruvate, 50 μg/ml gentamycin, 10 ng/ml of FSH and 10 ng/ml EGF in the presence of 5% fetal bovine serum (FBS). The addition of FSH, EGF and FBS was necessary for the synthesis of components of the extracellular matrix (ECM) by cumulus cells and for its organization around the cells. For this reason, the process is named cumulus expansion or mucification and it is functional to the extrusion of the cumulus oophorous at the time of ovulation and to the fertilization process. In all experiments, the incubation medium was supplemented with different concentrations of the tested NPs (between 0 and 200 μg/ml). The concentrations were chosen to perform a dose-response curve and to identify the range comprised between no-effect and toxicity; the concentrations used are comparable to those used in other in vitro published studies on other internal organs [63]. At the end of the culture time, the ability of cumulus complexes to respond to in vitro stimuli and expand was recorded. The level of expansion was classified by an expansion score from 0 to 4, with 0 being no expansion and 4 being the highest expansion [64]. Trypan blue staining was performed to evaluate cell viability. In details, 10 μl of a 0.4% Trypan blue stock solution (Sigma-Aldrich) were added to each 20 μl culture drop, incubated for 15 min and then rinsed with PBS. Images were acquired under a Leitz Diavert microscope connected to a camera.

### Preparation of ZnO NPs (NM-110 and NM-111) conditioned medium

To perform ZnO NP-conditioning, media were incubated with ZnO NM-110 and ZnO NM-111 for 16 h at 37 °C in a humidified atmosphere of 5% CO_2_, following the standard COC culture set-up, but in the absence of cumuli. Medium samples were then centrifuged at 10000 g for 1 h at 4 °C and the supernatants were collected and then used to culture COCs as reported above.

### Dispersion, preparation and characterization of uZnO and SiO_2_ZnO by DLS

uZnO and SiO_2_ZnO NPs were dispersed in 0.05% bovine serum albumin (BSA, Sigma Aldrich) in deionized water at a concentration of 1 mg/ml. The NP suspension was then sonicated on ice for 3 min at 800 watts (40% amplitude) using a 3 mm probe (Branson Digital Sonifier, Danbury, Connecticut, USA). After sonication, the batch dispersion was kept on ice and vortexed for 1 min right before use. The batch dispersion was diluted in the culture medium (EMEM 5% FBS) at a concentration of 5 μg/ml and 10 μg/ml and characterized by dynamic light scattering (DLS) in order to evaluate the hydrodynamic diameter (Z-average) and polydispersity index (PDI) values. DLS measurements of both batch dispersion and diluted samples were performed at 25 °C using a Zetasizer NanoS (Malvern Panalytical LtD). One milliliter of each sample was placed in a standard polystyrene cuvette and analyzed after 300 s in order to balance the sample. The instrument performs 10 repeated Z-average and PDI measurements of each sample. Measurements of diluted samples were carried out at 0, 3.5 and 16 h incubation time, and at both 5 μg/ml and 10 μg/ml.

### In vitro dosimetric analysis of uncoated ZnO and SiO_2_-coated ZnO NPs

The distorted grid (DG) model was used to simulate the particokinetics of uZnO and SiO_2_ZnO within the microdrops of medium that envelop the COCs [65]. Such simulation allows the calculation of the fraction of administered mass delivered to the surface of COC (f_D_) as a function of exposure time. Required parameters to run the simulation on MATLAB (MathWorks, Massachusetts, USA) include the height of the drop, the COC culture medium properties (density, viscosity), and particle properties (pristine particle density, agglomerate hydrodynamic size, and effective particle density in COC culture medium). The effective densities (ρ_eff_) of uZnO and SiO_2_ZnO were previously measured in a similar growth medium (EMEM 5% FBS) using the volumetric centrifugation method (VCM) as described by the authors [40, 66]. These ρ_eff_, including medium density and viscosity, were used as proxy values for the uZnO and SiO_2_ZnO NPs in EMEM supplemented with 5% FBS used in this study. Finally, the hydrodynamic particle agglomerate sizes (Z-average) were measured by DLS as described above.

### ICP-MS analysis of Zn^2+^ ions released by uZnO and SiO_2_ZnO NPs

The concentration of Zn^2+^ ions present in culture medium after 0, 3.5 and 16 h incubation of both uZnO and SiO_2_ZnO NPs was determined by ICP-MS. Zinc was measured in samples by a Thermo Scientific™ iCAP™ Q ICP-MS (Bremen, Germany) equipped with a quartz torch, a PFA concentric nebulizer and a quartz cyclonic spray chamber and Ni cones at interface. The detection of Zn, at the most abundant isotope (48.9%) of 64 m/z, using ICP-MS is typically affected by many polyatomic interferences such as ^32^S^16^O_2_^+^, ^48^Ca^16^O^+^, ^48^Ti^16^O^+^, ^36^Ar^14^N_2_^+^, ^31^P^16^O_2_^1^H^+^. These interferences were completely removed using kinetic energy discrimination (KED) and the QCell Collision/Reaction Cell system utilizing 100% helium (99.999% purity) at 4.8 mL/min as collision gas. Internal standardization with ^69^Ga was used to correct for instrumental drifts, and quantification was performed by the standard addition approach in order to account for matrix effects.

### RNA isolation from COCs and quantitative RT-PCR

After 3.5 h of culture with or without uZnO and SiO_2_ZnO NPs, COCs were washed three times in EMEM supplemented with 25 mM HEPES, 50 g/ml gentamycin and 1 mg/ml BSA and RNA was extracted using the TRIZOL Reagent (Roche Diagnostics GmbH, Mannheim, Germany), according to the manufacturer’s protocol. RNA quality was evaluated on agarose gels. Reverse transcription of mRNA and effective genomic DNA elimination was performed using the QuantiTect Reverse Transcription Kit (Qiagen, Hilden, Germany) following the manufacturer’s specifications. Gene expression was measured using Real Master Mix SYBR ROX (Eppendorf, Hamburg, Germany). qRT-PCR was performed using an Applied Biosystems 7300 Real Time PCR System (Applied Biosystems). Differences among gene expression were quantified using the ΔΔCt method with normalization to Gapdh. Specific primers for *Has2* (Hyaluronan synthase 2), *Ptx3* (Pentraxin 3), the HA receptor *CD44*, *Pfkp* (Phosphofructokinase, platelet) and *Grem1* (Gremlin 1) were designed using Primer Express software (Applied Biosystems in Life Technologies, Monza, Italy). Sequences are listed below:

*Has2*: 5′-GGCGGAGGACGAGTCTATGA-3′.

5′-TCTCAGGACACATAGAAACCTCTCA-3′;

*Ptx3*: 5′-CAGGAGAGCCGTGACGCGAG-3′.

5′-TGTTTCACAACCTGCGGGCAGC-3′.

*Cd44*: 5′-CCTTACCCACCATGGACCAA-3′.

5′-CCATACCTGCATGTTTCAAAACC-3′.

*Pkfp*: 5′- TGGAGCGGACTTCTGGAAGA-3′.

5′- ACTTCTGCACTGTGTCGTTATCG-3′.

*Grem1*: 5′- CGTGGCTCCCCAAATGTCT-3′.

5′- GGCCCACCCACCTTTCAC-3′.

*Gapdh*: 5′-AACTTTGGCATTGTGGAAGG-3′.

5′-CACATTGGGGGTAGGAACAC-3′.

### TEM/EDX analysis of COCs

After 3.5 h of culture in the presence of 10 μg/ml of uZnO and SiO_2_ZnO NPs, COCs were washed three times as above reported, fixed in 4% paraformaldehyde (PFA), embedded in agarose 2% and post-fixed in 2% osmium tetroxide. After washing with 0.1 M phosphate buffer, the sample was dehydrated by a series of incubations in 30, 50, and 70%, ethanol. Incubation steps in 95% ethanol, absolute ethanol, and propylene oxide continued dehydration. Samples were then embedded in Epon (Agar Scientific, Stansted Essex CM24 8GF United Kingdom). Eighty nm ultra-thin sections were mounted on copper grids and observed with Hitachi 7100FA transmission electron microscope (Hitachi, Schaumburg, IL, USA) for ultrastructural analysis.

To perform the analysis of elemental composition of the areas with electron-dense particles, unstained ultra-thin serial sections approximately 100-nm-thick were mounted on copper grids for microanalysis. EDX spectra were acquired with a Hitachi 7100FA transmission electron microscope (Hitachi, Schaumburg, IL, USA) and an EDX detector (Thermo Scientific, Waltham, MA, USA) at an acceleration voltage of 75 KeV and magnification of 12,000x. Spectra were semi-quantitatively analyzed by the Noran System Six k software (Thermo Scientific, Waltham, MA, USA) using the standardless Cliff–Lorimer k-factor method [67]. EDX microanalysis apparatus was calibrated using an X-ray microanalysis standard (Micro-Analysis Consultants Ltd., Cambridgeshire, UK).

### Statistical analysis

Gene expression values were expressed as mean ± standard error (SE) and differences between experimental groups were evaluated by one-way analysis of variance (ANOVA) using SigmaPlot 12.0 software. The ICP-MS analysis results were presented as percentage of the starting concentration and expressed as mean ± standard deviation (SD) and differences between experimental groups were evaluated by Student’s t-tests using SigmaPlot 12.0 software. Differences were considered to be statistically significant when *P* was less than 0.05.

## Supplementary Information

**Additional file 1 **: **Supplemental Fig. 1.** TEM analysis of **a, b** uZnO and **c, d** SiO_2_ZnO NPs. Reproduced with permission from Sotiriou et al. [22].

**Additional file 2 **: **Supplemental Fig. 2.** Delivered-to-cell mass fractions as a function of time for uZnO (red) and SiO_2_ZnO NPs (blue) at starting concentrations of 5 μg/ml (continuous line) and 10 μg/ml (dotted lines).

**Additional file 3 **: **Supplemental Fig. 3.** Phase contrast micrographs of COCs after 3.5 h of culture, the time of RNA extraction, **a** without NPs or in the presence of 10 μg/ml of **b** uZnO or **c** SiO_2_ZnO NPs. Scale bar = 100 μm.

## Data Availability

The datasets used and/or analyzed during the current study are available from the corresponding author on reasonable request.

## Abbreviations

NP: Nanoparticle
COC: Cumulus cell-oocyte complex
AgNP: Silver NP
TiO_2_NP: Titanium dioxide nanoparticle
AuNP: Gold nanoparticle
uZnO NP: Uncoated zinc oxide NP
SiO_2_ZnO NP: SiO_2_-coated zinc oxide NP
JRC: Joint Research Centre
E_i_: Expansion index
EDX: Energy Dispersive X-ray Spectroscopy
SSA: Specific surface area
f_D_: Mass fraction
HA: Hyaluronic acid
VENGES: Versatile Engineered Nanomaterial Generation System
TEM: Trasmission electron microscope
XRD: X-ray diffraction
XPS: X-ray photoelectron spectroscopy
BET: Brunauer–Emmett–Teller
EMEM: Minimum Essential Medium with Earle’s salts
FSH: Follicle stimulating hormone
EGF: Epidermal growth factor
FBS: Fetal bovine serum
ECM: Extracellular matrix
BSA: Bovine serum albumin
DLS: Dynamic light scattering
PDI: Polydispersity index
DG: Distorted grid
ρ_eff_: Effective density
VCM: Volumetric centrifugation method
ICP-MS: Inductively coupled plasma mass spectrometry
KED: Kinetic energy discrimination
qRT-PCR: Quantitative RT-PCR
Has2: Hyaluronan synthase 2
PTX3: Pentraxin 3
PFA: Paraformaldehyde
