# Supplementary figures and images for: Silica encapsulation of ZnO nanoparticles reduces their toxicity for cumulus cell-oocyte-complex expansion
Source: Part Fibre Toxicol. 2021 Sep 3;18:33. doi: 10.1186/s12989-021-00424-z (PMC8414698; doi:10.1186/s12989-021-00424-z)

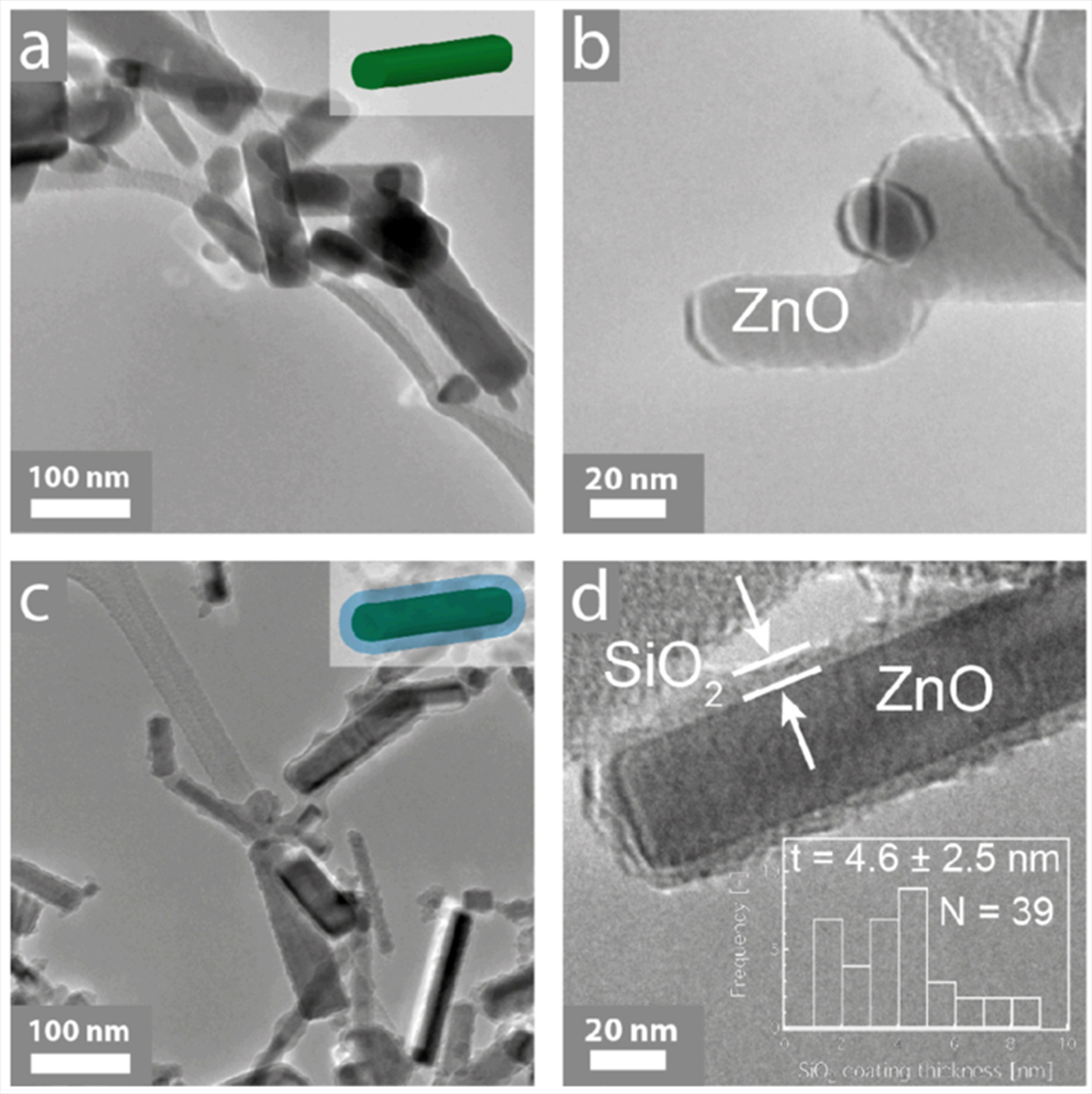

Supplement: Supplementary file 1 — Additional file 1 : Supplemental Fig. 1. TEM analysis of a, b uZnO and c, d SiO2ZnO NPs. Reproduced with permission from Sotiriou et al. [22]. [file 12989_2021_424_MOESM1_ESM.tif]

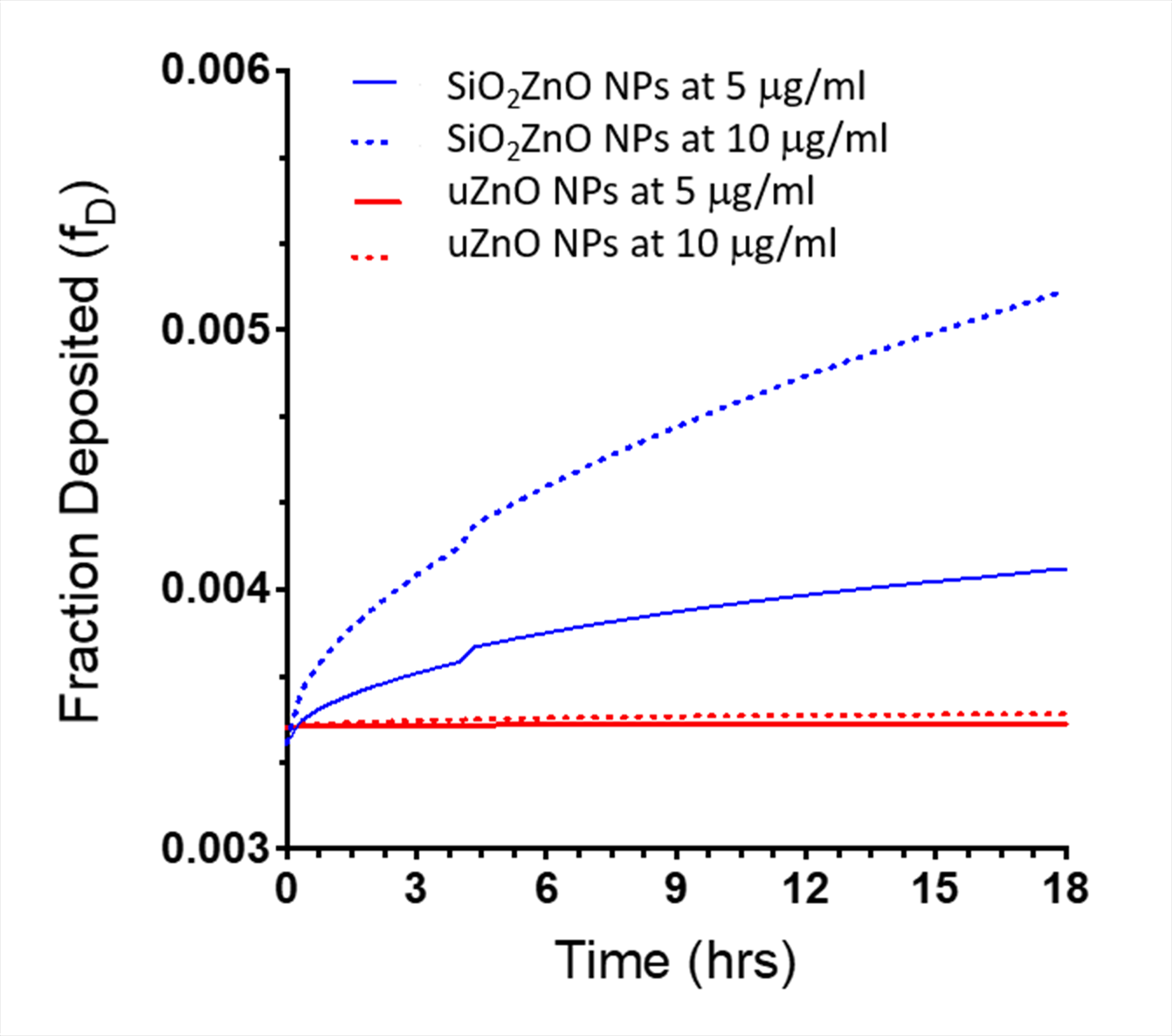

Supplement: Supplementary file 2 — Additional file 2 : Supplemental Fig. 2. Delivered-to-cell mass fractions as a function of time for uZnO (red) and SiO2ZnO NPs (blue) at starting concentrations of 5 μg/ml (continuous line) and 10 μg/ml (dotted lines). [file 12989_2021_424_MOESM2_ESM.tif]

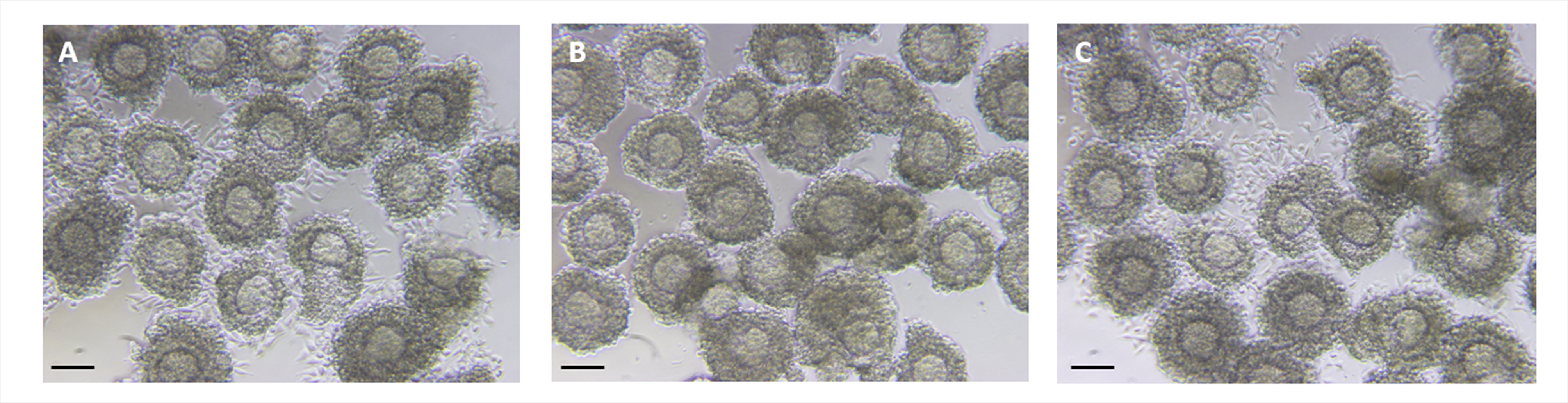

Supplement: Supplementary file 3 — Additional file 3 : Supplemental Fig. 3. Phase contrast micrographs of COCs after 3.5 h of culture, the time of RNA extraction, a without NPs or in the presence of 10 μg/ml of b uZnO or c SiO2ZnO NPs. Scale bar = 100 μm. [file 12989_2021_424_MOESM3_ESM.tif]
